# Measuring dichotomous outcomes using risk ratios, odds ratios, and the risk difference: A tutorial

**DOI:** 10.1002/cesm.12022

**Published:** 2023-07-24

**Authors:** Rachel Richardson, Kerry Dwan, Afroditi Kanellopoulou

**Affiliations:** ^1^ Evidence Production and Methods Department Cochrane, London UK; ^2^ Liverpool School of Tropical Medicine Liverpool UK

## Abstract

This article provides guidance on measuring dichotomous outcomes, focusing on the three main effect measures that you can use. We explain how to calculate and interpret these measures. We have also developed a micro learning module so that you can practice calculating risk ratios, odds ratios, and risk differences.

Dichotomous outcomes micro learning module

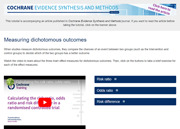

## 1 INTRODUCTION

In this article, we look at risk ratios (RR), odds ratios (OR), and the risk difference (RD); what they are, how to interpret them, and when they should be used.

The RR, the OR, and the RD are used to compare the occurrence of an event in two groups for dichotomous outcomes. For example, a recent Cochrane review compared oral misoprostol to placebo for induction of labour and reported whether the women in each group went on to have a caesarian section, or not [1].

## 2 RISK RATIO

The RR provides a measure of how much higher or lower the risk of the event happening in the intervention group is, compared to the risk of the same event happening in the control group. Risk might sound like a complicated term but it is actually just the probability of an event happening.

Example: Trial A is interested in whether patients were readmitted to hospital within 30 days of an operation (procedure X) and compared this with patients who had a different operation (procedure Y). One hundred out of 200 patients who had procedure X were readmitted within 30 days. The risk (or probability) of this event occurring is 100 divided by 200, or 0.5. One hundred and fifty patients out of 200 patients who had procedure Y were readmitted within 30 days. The risk (or probability) of this event occurring is 150 divided by 200 or 0.75.

$$\begin{array}{lll} \text{Ratio}\;\text{of}\;\text{these}\;\text{risks} & = & \text{risk}\;\text{with}\;\text{procedure}\;X\;\text{divided}\;\text{by}\;\text{the}\;\text{risk}\;\text{with}\;\text{procedure}\;Y\\ & = & 0.5/0.75\\ & = & 0.67 \end{array}$$

We can also work out how much procedure X reduces the risk of readmission compared to procedure Y. If an event is certain to happen, we say that the risk is 1. Procedure X reduces this risk by 0.67. Therefore procedure X reduces this risk, as follows:

$$\begin{array}{lll} \text{Risk}\;\text{of}\;\text{being}\;\text{readmitted} & = & 1.00\;‒\;0.67\\ & = & 0.33 \end{array}$$

This means that if patients have procedure X, their risk of being readmitted is **reduced** by 33% compared to their risk if they had procedure Y. We can also express this as a probability—the probability of them being readmitted is reduced by 33%.

On the other hand, if 140 out of 200 patients who had procedure X (risk: 140/200 = 0.7) and 120 of 200 patients who had procedure Y (risk: 120/200 = 0.6) were readmitted within 30 days, then the RR equals 0.7/0.6 = 1.17. This means that if patients have procedure X, their risk of being readmitted is **increased** by 17% compared to their risk if they have procedure Y. When the risk in the intervention group is the same as the risk in the control group, the RR will be 1, and we can say that the risks are the same for both groups.

## 3 ODDS RATIO

The OR provides a measure of how much higher or lower the odds of the event happening in the intervention group are compared to the odds of the same event happening in the control group. Odds may also sound like a complicated term, but it merely refers to the probability of something happening compared to the probability of it not happening. If the odds of a horse winning the Kentucky Derby are 7 to 2 against, this means that over nine races it would be predicted to win twice and lose seven times.

*Example*: Let's go back to our hypothetical example of procedure X versus procedure Y. If 100 out of 200 patients who had procedure X were readmitted within 30 days, the odds of readmission would be 100/100 or 1. In betting terminology, this would be “evens”: the chances of being readmitted and not being readmitted are the same. If 150 out of 200 patients who had procedure Y were readmitted, the odds of being readmitted are 150/50, or 3 to 1.

$$\begin{array}{lll} \text{Ratio}\;\text{of}\;\text{these}\;\text{odds} & = & \text{odds}\;\text{with}\;\text{procedure}\;X\;\text{divided}\;\text{by}\;\text{the}\;\text{odds}\;\text{with}\;\text{procedure}\;Y\\ & = & 1/3\\ & = & 0.33 \end{array}$$

This means that the odds of being readmitted after procedure X are reduced compared to procedure Y. A patient who has procedure X has the same chance of being readmitted versus not being readmitted, whereas three out of four patients who have procedure Y will be readmitted.

In the second scenario, if 140/200 procedure X patients (odds: 140/60 = 2.3) and 120/200 procedure Y patients (odds: 120/80 = 1.5) are readmitted within 30 days, then the OR equals 2.3/1.5 = 1.6.

As with RR, if the odds in an intervention group are the same as the odds in a control group, the OR will be 1, and we can say that the odds are the same in both groups.

## 4 RISK DIFFERENCE

The RD is an absolute rather than a ratio measure and tells us the difference between the probabilities of the event occurring in the two groups. In the first scenario above the risk of readmission in the procedure X group is 100/200 or 0.5. The risk for the procedure Y group is 150/200 or 0.75. The RD is 0.5–0.75, which equals −0.25. This means that for every hundred people, 25 fewer will be readmitted with procedure X compared to procedure Y.

## 5 WHAT DO CONFIDENCE INTERVALS MEAN?

Effect estimates such as risk and OR are calculated from data that have been taken from a sample of the whole population. This means that we cannot be sure that our estimate is the true value: the confidence intervals (CIs) give us a “margin of error.” For example, a RR of 0.67 with 95% CIs ranging from 0.57 to 0.78 means (broadly speaking) that we can be 95% certain that the true effect estimate lies between these two values. It is also possible to calculate CIs for different values, for example, 90% or 99%.

## 6 DIFFERENCES BETWEEN RR AND OR

As we can see from the examples above, the effect estimates produced by RR and ORs can differ markedly because of the different ways in which they are calculated.

When events are infrequent, then risk and odds appear similar. However, when events occur more frequently, the odds appear very different to the risk. These differences affect the calculations for the RR and OR. Going back to the previous example: if the risk of readmission for people undergoing procedure X and procedure Y was low (e.g., 15 out of 200 people in the procedure X group and 10 of out of 200 people in the procedure Y group then the RR would be 0.075/0.05 = 1.5. The OR in the same scenario would be 0.08/0.05 = 1.6. However, if the risk of readmission was much higher (e.g., 150 people in the procedure X group and 100 in the procedure Y group) then the RR would be 0.75/0.5 = 1.5 whilst the OR would be 3/1 = 3.0.

## 7 WHICH MEASURE SHOULD I USE AND WHEN?

The OR can produce more “extreme” values than the RR. If the OR is interpreted as if it were a RR, this can lead to misinterpretation. For this reason, it will often be better to use the RR as the preferred effect measure in systematic reviews. However, OR have properties that can make them easier to handle from a statistical point of view.

We often see that meta‐analyses including OR have higher heterogeneity compared to meta‐analyses of RR. Nevertheless, authors should never rely on the magnitude of heterogeneity to choose which type of estimate works best.

The RD can be helpful when working out the “real world” implications of choosing one intervention rather than another. For example, the knowledge that procedure X is likely to prevent readmission for 25 out of every 100 patients compared to procedure Y can help clinicans decide which procedure to implement.

The key for reviewers is to prespecify which measures will be used and to handle and interpret their chosen type of effect measure correctly.

## 8 FURTHER READING AND ONLINE CONTENT

More information on calculating and using risk and OR, as well as other dichotomous outcomes can be found in Chapter 6.4 of The Cochrane Handbook for Systematic Reviews of Interventions [2].

Cochrane Training has produced a microlearning module about measuring dichotomous outcomes to accompany this article [3].


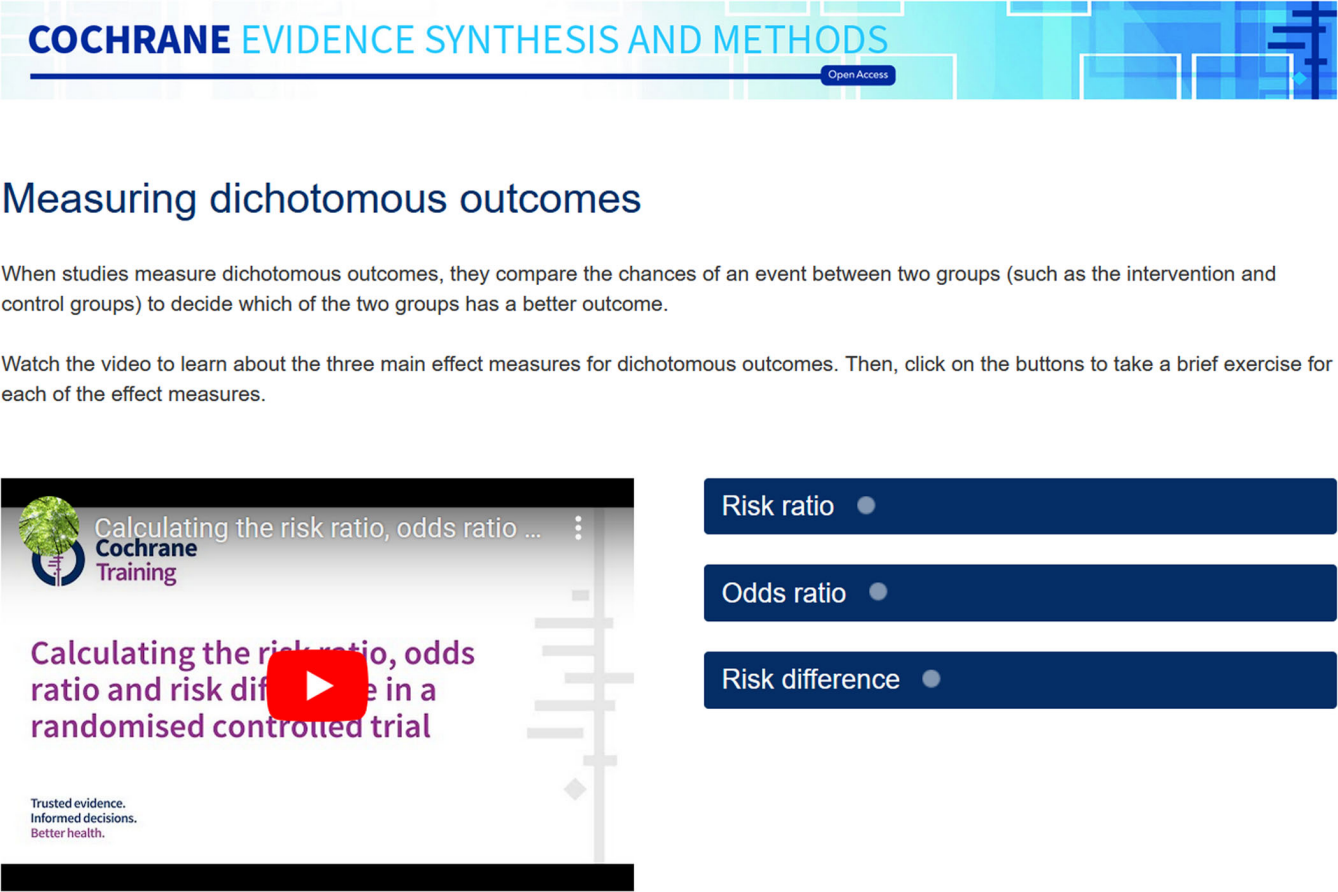


Professors Bland and Altman discuss the properties of the OR in more detail in their Statistics Notes series, published in the BMJ [4].

## AUTHOR CONTRIBUTIONS

**Rachel Richardson**: Conceptualization; methodology; project administration; writing—original draft; writing—review and editing. **Kerry Dwan**: Conceptualization; writing—review and editing. **Afroditi Kanellopoulou**: Conceptualization; writing—original draft.

## 1 PEER REVIEW

The peer review history for this article is available at https://www.webofscience.com/api/gateway/wos/peer-review/10.1002/cesm.12022.

## Data Availability

Data sharing not applicable to this article as no data sets were generated or analyzed during the current study.
